# The prognostic value of the histological shape of tumor negative sentinel nodes in breast cancer

**DOI:** 10.3389/fimmu.2023.1258641

**Published:** 2023-10-30

**Authors:** Loes F. S. Kooreman, Sabine Dieleman, Sander M. J. van Kuijk, Axel zur Hausen, Marjolein L. Smidt, Heike I. Grabsch

**Affiliations:** ^1^ Department of Pathology, GROW School for Oncology and Reproduction, Maastricht University Medical Center+, Maastricht, Netherlands; ^2^ GROW School for Oncology and Reproduction, Maastricht University Medical Center+, Maastricht, Netherlands; ^3^ Department of Surgery, Maastricht University Medical Center+, Maastricht, Netherlands; ^4^ Department of Clinical Epidemiology and Medical Technology Assessment, Maastricht University Medical Center+, Maastricht, Netherlands; ^5^ Pathology and Data Analytics, Leeds Institute of Medical Research at St. James’s, University of Leeds, Leeds, United Kingdom

**Keywords:** tumor draining lymph node, sentinel lymph node, breast cancer, prognosis, morphometry, histopathology, tumor infiltrating lymphocytes

## Abstract

**Introduction:**

Sentinel lymph node (SLN) metastasis is an important predictor of prognosis in breast cancer (BC) patients, guiding treatment decisions. However, patients with the same BC subtype and tumor negative SLN (SLNneg) can have different survival outcomes. We hypothesized that the host anti-tumor immune reaction in SLNneg is important and results in morphometrically measurable changes in SLN size or shape which are related to patient prognosis.

**Methods:**

Surface area, circumference, long axis and short axis were histologically measured in 694 SLNneg from 356 cases of invasive BC and 67 ductal carcinoma *in situ* cases. The area occupied by fat was categorized as less or more than 50%. The long to short axis (L/S) ratio was calculated. The relationship between SLNneg morphometries and clinicopathological variables like tumor-infiltrating lymphocytes (TILs) within the primary tumor, as well as prognosis at 10 years follow up were analyzed.

**Results:**

The mean SLNneg surface area was 78.7mm^2^, circumference 40.3mm, long axis 13.1mm, short axis 8.2mm and L/S ratio 1.7. Larger surface area, long axis and short axis, including age >55 years were associated with higher body mass index (BMI) and SLN fat over 50% (p<0.003). In invasive BC, a high SLNneg L/S ratio (≥1.9) was related to poorer disease-free (HR=1.805, 95%CI 1.182-2.755, p=0.006) and overall (HR=2.389, 95%CI 1.481-3.851, p<0.001) survival. A low SLNneg L/S ratio (<1.9) was associated with high TILs in the primary BC (≥10%) (p=0.005). However a high TIL count was not of prognostic relevance.

**Conclusions:**

This is the first study to suggest that morphometric characteristics of axillary SLNneg, like L/S ratio, could be used to predict prognosis in patients with SLNneg invasive BC of all subtypes. The association between low L/S ratio and high TILs suggest that SLN shape is related to immunological functioning of the SLN and could be used in addition to TIL evaluation. Regarding the dubious role of TILs in hormone receptor positive breast cancer, SLNneg morphometry to gain information about host immune status could especially be of benefit in this subtype. Further studies are warranted to better understand the underlying biological mechanisms.

## Introduction

Breast cancer (BC) is the most frequently diagnosed cancer in women worldwide (1). Current therapeutic interventions are based on molecular BC subtype, grade and disease stage combined with patient characteristics (2, 3). Regional tumor draining lymph nodes (LNs) are most commonly located in the axilla and are often the initial site of LN metastasis. Without reasonable clinical suspicion of LN metastasis, the sentinel lymph node (SLN), which is the presumed first tumor draining LN, is resected at the time of primary tumor resection (4). In current routine BC diagnostics, SLNs are histologically assessed for the presence of metastasis. No details are collected on the SLN microarchitecture (such as reactive germinal centers) and/or morphometrical features (such as size) by the pathologist. More importantly, BC patients with negative SLNs (SLNnegs) exhibit differences in survival, even after correction for tumor type, size and grade (5).

The host anti-tumor immune response plays an essential role in eradicating cancer cells (6). However, primary tumor infiltrating immune cells can either have pro-tumorigenic or anti-tumorigenic effects. In BC, a high number of primary tumor-infiltrating lymphocytes (TILs) have been related to better response to neoadjuvant therapy in all BC subtypes and to a better prognosis in triple negative breast cancer (TNBC) and HER2 positive BC patients (7, 8). The prognostic value of high TILs in hormone receptor positive BC is low. Many recent BC studies focused on investigating the immune response in the primary tumor (6, 9, 10). Only few studies investigated the relationship between immune reaction in tumor-draining negative LNs, focusing on sinus histiocytosis and germinal centers and survival in BC (11). A high degree of sinus histiocytosis in axillary LNs, irrespective of tumor presence, was found to be associated with increased overall survival (OS) (11). Another study showed an association between a high number of germinal centers in tumor free axillary LNs, high number of TILs in the primary tumor and better disease free survival (DFS) and OS in TNBC patients with metastasis in one of the other resected LNs (12). In one study examining axillary lymph node dissection (ALND) in BC patients, higher numbers of germinal centers and a higher number of adipocytes within the LN were related to an increased LN size (13). Similarly, a relationship between increased LN size, higher number of germinal centers, lymphocyte predominance (also referred to as paracortical hyperplasia), and higher intranodal fat content has been reported (13).

However, to the best of our knowledge, no study is available investigating morphometric characteristics of SLNnegs, such as surface area and length, and their relationship with prognosis in BC patients. We hypothesized that larger SLNneg size reflects extensive host anti-tumor immune activation and is associated with better patient prognosis. The aim of this study was to histologically determine the morphometrical characteristics (surface area, circumference, long axis, short axis, long/short axis ratio and extent of intranodal fat) of axillary SLNnegs in a retrospective cohort of BC patients and investigate their relationship with clinicopathological data, survival and primary tumor immune status *via* TILs.

## Materials and methods

### Patients

Patients with ductal carcinoma *in situ* (DCIS) or invasive breast cancer (iBC) who underwent sentinel lymph node (SLN) surgery at Maastricht University Medical Center+ (MUMC+), Maastricht, the Netherlands, between January 2007 and December 2011 were retrospectively identified from the pathology database. Patients with metastasis or isolated tumor cells in any of the resected lymph nodes, multifocal primary tumors or patients receiving neoadjuvant treatment were excluded.

Clinicopathological data were retrieved from BC cases. Information on tumor laterality, type of surgery, resection margin status and date of BC diagnosis were extracted from the pathology laboratory information system. In addition, data on histological subtype, ER, PR, and HER2 expression status, tumor size, tumor grade, presence of lymphovascular space invasion, and presence of associated DCIS was extracted and reviewed for all iBC cases. Primary tumor-infiltrating lymphocytes (TILs) were determined according to the recommendations from the International TILs Working Group (14) as percentage area of stroma covered with TILs. For DCIS cases, DCIS grade and diameter were extracted and a cutoff for DCIS size of 40mm was considered as risk factor for local recurrence as reported by Sagara (15). Information on patient characteristics (sex, age, body mass index (BMI), presence of previous malignancy), treatment (adjuvant chemotherapy, adjuvant hormonal therapy and adjuvant radiotherapy) and clinical outcome data (last follow up date, survival status, occurrence of recurrent local disease or metastasis) were retrieved from the MUMC+ electronic patient database. Treatment adequacy was determined using international guidelines as previously described. Events of epithelial skin cancers were not included as history of malignancy. Age of diagnosis older than 55 years was used as a surrogate marker for postmenopausal women (16). This cut off was used to form age groups. The study was approved by the local ethical committee (METC 2021-2603) and patient informed consent was waived.

### Delineation of sentinel lymph nodes

**Figure 1:**
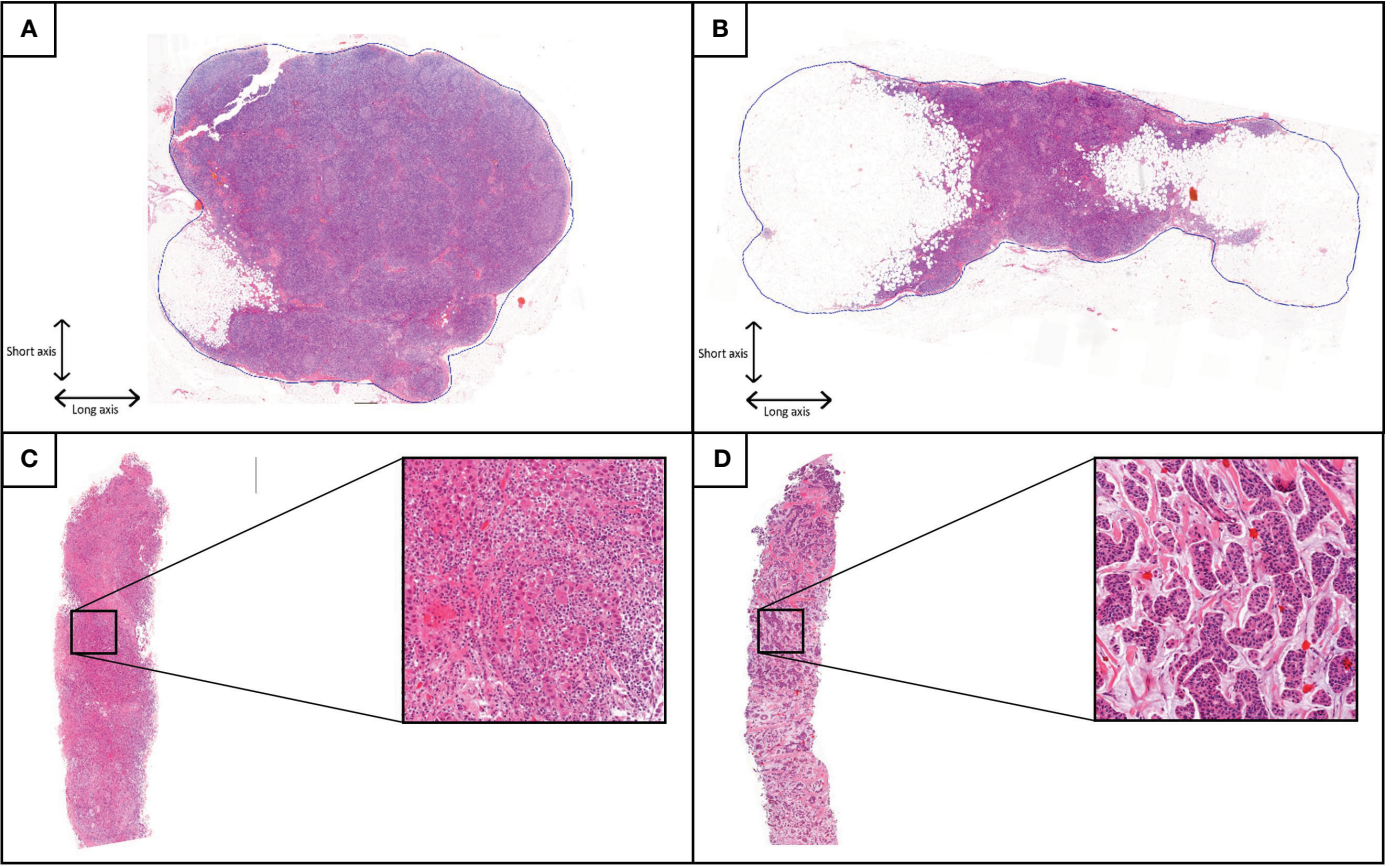
Annotations of two sentinel lymph node sections with paired breast needle biopsies. Example annotations of tumor negative sentinel lymph node (SLNneg) sections, with the blue line indicating circumference at the capsula of the SLNneg. **(A)** SLNneg with surface area of 92.8mm2, circumference 37.4mm, long axis 11.6mm, short axis of 9.9mm, L/S ratio of 1.2 and fat percentage less than 50%. **(B)** SLNneg with surface area of 119.5mm2, circumference 45.0mm, long axis 18.5mm, short axis 7.9mm, L/S ratio of 2.4 and fat percentage over 50%. **(C)** Paired breast core needle biopsy of SLNneg from A, with 80% stromal tumor-infiltrating lymphocytes. **(D)** Paired breast core needle biopsy of SLNneg from B, with 0% stromal tumor-infiltrating lymphocytes.

All hematoxylin and eosin (H&E) slides of SLNnegs from all cases were retrieved from the pathology archive at MUMC+ and scanned at 40x magnification using 3DHistech Panoramic p1000 scanner. Using Medical Image Manager (MIM software, HeteroGenius Ltd. Leeds, UK), digital images were reviewed, and lymph nodes were segmented by an expert breast pathologist (LK) blinded to patient and tumor characteristics. All SLNnegs levels were individually segmented on all slides. If a LN capsule was visible, this was regarded as the border of the LN, **Figure 1**. If such a capsule was not visible, for instance in case of excessive fat containing LNs, the border of the LN was regarded as where lymphocytes ceased to be interspersed between adipocytes. The characteristics of the SLNneg with the largest surface area were used for analysis.

### Calculation of morphometrical characteristics

After manually segmenting the LN, morphometrical characteristics were calculated by the image analysis software, including surface area, circumference, long axis, and short axis, **Figure 1**. LN shape was defined by the ratio between the length of the long axis and that of the short axis (L/S ratio). Perfectly round LNs have a L/S ratio of 1.0. The percentage area of the LN containing fat was estimated visually and dichotomized at 50% (< 50%: non-fatty nodes, ≥ 50% fatty nodes).

### Statistical analyses

From every case, the SLNneg with the largest surface area was used for analyses and normal distribution was verified using histogram. SLNneg morphometries were expressed as mean ± standard deviation (SD). The relationship with clinicopathological characteristics and treatment modalities was analyzed using one-way analysis of variance (One-way ANOVA). The ANOVA assumptions, comprising normality, equality of variances, and homoscedasticity, were verified for the entire group and individual subgroups. Associations between extent of fat within nodes, age and BMI were tested with Pearson’s chi-square test, and a comparison between multiple groups were made using general linear model and Bonferroni tests. TILs were considered as continuous and were dichotomized into high or low groups based on the mean.

The primary endpoint of this study was 10-years disease free survival (DFS) and overall survival (OS), measured from time of diagnosis. Differences in survival were assessed using Kaplan-Meier plots and tested using the log-rank test. Cox proportional hazards regression models were used for univariate and multivariate analysis of prognostic factors. For iBC, univariate cox regression analysis was performed for variables including age, BMI, tumor size, tumor grade, tumor stage, histological subtype, ER expression, PR expression, HER2 status, breast cancer subtypes based on immunohistochemistry (ER+/HER2-, HER2+, triple negative), positivity of resection margins, lymphovascular space invasion, perineural growth, presence of previous malignancy, treatment, adequacy of treatment, and TILs. For DCIS, univariate cox regression analysis was performed for age, BMI, DCIS size, and DCIS grade. Variables significant in univariate cox regression analysis were included in the multivariate cox regression analysis. For categorical variables, proportionality of risks was assessed visually using Kaplan-Meier plots. For continuous variables, we assessed whether there was an association between time and the scaled Schoenfeld residuals. A receiver operating characteristic (ROC) curve was used to define a cutoff for survival analysis for linear prognostic significant morphometrics of the SLNneg. All p-values were two-sided and considered significant if less than 0.05. IBM SPSS version 27.0 was used to perform statistical analyses. The Schoenfeld residuals are performed with R, version 4.2.2. This study adhered to the STROBE guidelines, **Supplementary Table 1** (17).

## Results

### Study cohort characteristics

From the pathology database, 744 BC patients where included. After exclusion of patients with metastasis or isolated tumor cells in any of the resected lymph nodes (n=274), multifocal primary tumors (n=34) or receiving neoadjuvant treatment (n=13), 409 female patients remained for analysis in the current study. Clinicopathological data were retrieved from 67 DCIS cases and 356 iBC cases. Clinicopathological characteristics of iBC are summarized in **Table 1**. All patients underwent primary BC resection with SLN resection. Bilateral disease occurred in 14 patients, contributing to a total of 423 cases.

**Table 1:** Clinicopathological characteristics of invasive breast cancer patients.

Variable	No. of patients (N=356)
Age	
Mean (SD)	60.3 (11.3)
BMI (kg/m^2^)	
≤ 25 26-30 30	114 (32.0%) 94 (26.4%) 55 (15.4%)
Type of surgery	
Lumpectomy Mastectomy	205 (57.6%) 151 (42.4%)
Histological subtype^1^	
NST Lobular Other	266 (74.7%) 28 (7.9%) 58 (16.3%)
T stage^1^	
T1 T2 T3	283 (79.5%) 67 (18.8%) 2 (0.6%)
Tumor grade^1^	
I II III	99 (27.8%) 149 (41.9%) 102 (28.7%)
Margin status	
Positive Negative	17 (4.8%) 339 (95.2%)
ER^1^	
Positive Negative	286 (80.3%) 59 (16.6%)
PR^1^	
Positive Negative	233 (65.4%) 111 (31.2%)
HER2^1^	
Positive Negative	38 (10.7%) 285 (80.1%)
Combined receptor status^1^	
ER+/HER2-^1^ HER2+ TNBC	242 (74.9%) 38 (11.8) 57 (13.3%)
TNBC^1^	
Yes No	43 (12.1%) 299 (78.7%)
TILs	
< 10% ≥ 10%	257 (72.2%) 95 (26.7%)
Therapy adequacy^1^	
Adequate Inadequate	288 (81%) 67 (19%)
Radiotherapy^1^	
Yes No	206 (57.9%) 148 (41.6%)
Systemic therapy^1^	
Yes No	165 (46.3%) 188 (52.8%)
Malignancy in history^2^	
Yes No	42 (11.8%) 314 (88.2%)

SLNneg, tumor negative sentinel lymph node. NST, invasive breast cancer no special type. ILC, invasive lobular carcinoma. TNBC, triple negative breast cancer. BMI, body mass index.

^1^indicates that there were missing data but at least 90% data were available, except for BMI where only 72,5% of data were available.

^2^indicates history of cancer other than epithelial skin cancer.

In total, of 423 cases, 694 SLN were resected. SLNs were handled in the pathology department following Dutch national guidelines which includes cutting the SLN at 3 levels and performing cytokeratin immunohistochemistry if appropriate (18). All SLNs from every patient were confirmed to be tumor negative (SLNneg) and without isolated tumor cells (18). The mean ± SD number of SLNnegs per patient was 1.6 ± 0.8. The median age at diagnosis was 59.9 years (range 25-87 years). Fifty-eight cases (13.7%) had died at the end of follow-up and 40 cases (9.5%) developed BC metastasis or recurrence during the follow-up period.

### SLNneg morphometry and clinicopathological variables

In total, 694 SLNnegs were examined at multiple levels, resulting in 3081 scanned slides. On each slide, multiple sections of the same SLNneg were present and segmented, resulting in a total of 6368 segmentations. The mean ± SD of the surface area was 78.7 ± 54.2mm^2^, mean ± SD circumference 40.3 ± 16.2mm^2^, mean ± SD long axis 13.1 ± 4.9mm^2^, and mean ± SD short axis 8.2 ± 3.0mm^2^. SLNnegs had a mean ± SD L/S ratio of 1.7 ± 0.5. A long axis which is 1.7 times longer than the short axis suggests a bean-like or elongated shape. SLNnegs with a smaller L/S ratio indicates a more rounded shape, see **Figure 1**. There was no significant difference in SLNneg morphometric measurements between age groups, laterality or cases with DCIS compared to those with iBC, **Supplementary Table 2**.

Of all 423 SLNneg cases, a total of 73 cases (17.3%) contained more than 50% fat, see **Figure 1**. The mean surface area, circumference, long axis and short axis were significantly increased in fatty SLNnegs compared to non-fatty SLNneg (p<0.001), **Supplementary Table 2**. The L/S ratio did not differ between fatty and non-fatty SLNnegs. Cases with a higher BMI had SLNneg with increased surface area (p=0.003), long axis (p=0.014) and short axis (p=0.024), **Table 2**. Fatty SLNnegs were more common in cases older than 55 years (p<0.001), but were not significantly associated with BMI (p=0.301), **Table 2**.

**Table 2:** Association between age, BMI and presence of more than 50% intranodal fat.

		n	Age (years)			SLN Fat content		
			<55y	≥55	p-value	<50%	≥50%	p-value
BMI	≤25	138 (44%)	56 (40%)	82 (60%)	0.020*	114 (83%)	24 (17%)	0.301
	26-30	113 (36%)	27 (24%)	86 (76%)		92 (81%)	21 (19%)	
	>31	60 (19%)	20 (33%)	40 (66%)		44 (73%)	16 (27%)	
Age	<55y	134 (32%)				122 (91%)	12 (9%)	0.002*
	≥55y	289 (68%)				228 (79%)	61 (21%)	

SLNneg, tumor negative sentinel lymph node. BMI, body mass index. y, years.

p-values calculated with Pearson Chi-Square test. *Statistically significant p-values.

BMI of ≤25 was significantly less frequent in age group ≥55 using GLM and Bonferroni.

In iBC cases, a shorter long axis was significantly associated with progesterone receptor (PR) negative primary BC (p=0.044). There was no significant association between surface area, circumference, long axis, short axis or L/S ratio with tumor stage, histological subtype, tumor grade, ER status, HER2 status, TNBC subtype or lymphovascular space invasion, **Supplementary Table 3**. There was no significant difference in SLNneg morphometries between iBC and DCIS cases. In DCIS cases, no significant associations were found between SLNneg morphometrics and DCIS grade or DCIS size >40mm, **Supplementary Table 4**.

TILs were analyzed in 352 cases with iBC, with a median TILs percentage of 2.0%. High TILs were defined as higher than the mean (9%) and rounded at ≥10% for clinical applicability, with 95 cases (27%) having 10% or higher TILs. No patient had a TILs percentage between 9% and 10%. High TILs were associated with larger short axis (p=0.042) and lower L/S ratio (p=0.005) of the SLNneg, see **Figure 1**. No associations were found between high TILs and surface area, circumference, long axis and LN fat content, **Supplementary Table 3**.

### SLNneg morphometry and survival

**Figure 2:**
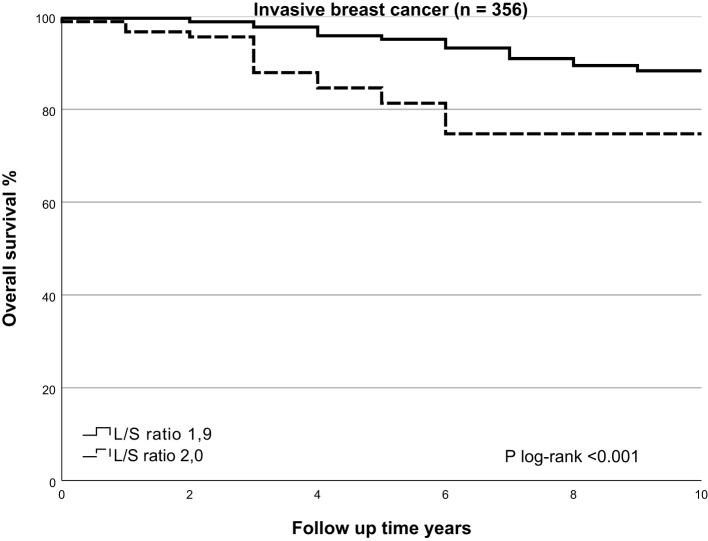
Kaplan Meier of overall survival by SLNneg L/S ratio in invasive breast cancer. Using the Kaplan-Meier curve to compare overall survival prospects for the high-risk SLNneg L/S ratio and low-risk SLNneg L/S ratio in invasive breast cancer patients. The high-risk and low-risk groups were identified using a receiver operating characteristic (ROC) curve. High-risk SLNneg L/S ratio >1.9 versus low-risk SLNneg L/S ratio ≤1.9; log-rank test, p<0.001.

**Figure 3:**
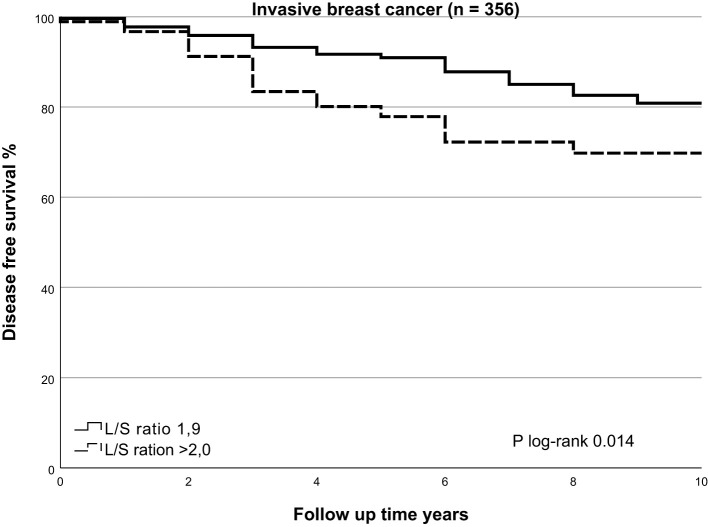
Kaplan Meier of disease free survival by SLNneg L/S ratio in invasive breast cancer. Using the Kaplan-Meier curve to compare disease free survival prospects for the high-risk SLNneg L/S ratio and low-risk SLNneg L/S ratio in invasive breast cancer patients. The high-risk and low-risk groups were identified using a receiver operating characteristic (ROC) curve. High-risk SLNneg L/S ratio >1.9 versus low-risk SLNneg L/S ratio ≤1.9; log-rank test, p=0.014.

In univariate analysis, cases with higher L/S ratio had poorer 10-years OS and DFS (HR=2.195, 95%CI 1.421-3.390, p<0.001 and HR=1.724, 95%CI 1.162 – 2.559, p=0.007, respectively), **Table 3** for main results and **Supplementary Table 5** for complete results. A high L/S ratio remained a significant predictor of poor OS and DFS in multivariate analysis adjusting the model for age, PR expression, ER+/HER2-, HER2+ and TNBC subtype, treatment adequacy, history of malignancy and tumor size with cutoff at 10mm OS (HR=2.357, 95%CI 1.458-3.812, p<0.001), DFS (HR=1.810, 95%CI 1.187-2.760, P=0.006). There was no lost to follow up for overall survival. The mean follow up for disease free survival was 8.4 years ± SD 2.6. The L/S ratio cutoff value identified using a ROC curve approach was 1.9 [sensitivity, 42.6%; specificity, 77.5%] for DFS and OS. A L/S ratio above 1.9 is related to a poorer 10-years overall survival (p<0.001), Kaplan-Meier **Figure 2** and **Table 4** and disease-free survival (p=0.014), Kaplan-Meier **Figure 3** and **Table 5**. Clinicopathological characteristics stratifying cases by high and low L/S ratio using the 1.9 cutoff are summarized in **Supplementary Table 6**. The presence of high TILs was the only difference between these groups, but high TILs was not significant related to prognosis in univariate survival analysis. In contrast to the findings for the L/S ratio, no significant survival difference was found for the long or short axis separately.

**Table 3:** Univariate and multivariate survival analyses of SLNneg in patients with invasive breast cancer.

	Disease free survival				Overall survival			
	Univariate		Multivariate		Univariate		Multivariate	
	HR (95% CI)	*p*-value	HR (95% CI)	*p*-value	HR (95% CI)	*p*-value	HR (95% CI)	*p*-value
Age (continuous, unit = 1 year)	1.060 (1.037-1.083)	<0.001*	1.061 (1.037-1.086)	<0.001*	1.076 (1.048-1.105)	< 0.001*	1.072 (1.043-1.102)	<0.001*
TILs (continuous, unit = 1%)	0.996 (0.982-1.011)	0.625	–	–	0.998 (0.982-1.014)	0.783	–	–
TILs cut-off (<10%) vs (≥10%)	1.105 (0.667-1.829)	0.699	–	–	0.756 (0.398-1.437)	0.394	–	–
Tumor size (≥10mm vs < 10mm)	1.471 (0.875-2.473)	0.145	–	–	3.291 (1.487-7.281)	0.003*	3.134 (1.227-8.006)	0.017*
PR expression (positive vs negative)	0.523 (0.332-0.823)	0.005*	0.571 (0.317-1.026)	0.061	0.524 (0.307-0.895)	0.018*	0.629 (0.301-1.315)	0.218
ER+/HER2- ER-/HER2+ TNBC	Ref 1.118 (0.529-2.364) 1.969 (1.102-3.516)	Ref 0.769 0.022*	Ref 1.142 (0.513-2.541) 1.744 (0.839-3.624)	Ref 0.746 0.136	Ref 1.195 (0.500-2.859) 2.249 (1.159-4.368)	Ref 0.688 0.017*	Ref 1.254 (0.472-3.334) 2.054 (0.851-4.958)	Ref 0.650 0.110
Treatment adequacy (yes vs no)	0.554 (0.335-0.916)	0.021*	0.727 (0.413-1.278)	0.268	0.516 (0.288-0.925)	0.026*	0.819 (0.408-1.645)	0.575
Malignancy in history^1^ (yes vs no)	2.328 (1.340-4.043)	0.003*	1.553 (0.847-2.846)	0.154	2.638 (1.413-4.925)	0.002*	1.789 (0.883-3.626)	0.106
Surface mm^2^ (continuous, unit = 1 mm^2^)	0.998 (0.993-1.002)	0.298	–	–	1.001 (0.996-1.006)	0.668	–	–
Circumference mm (continuous, unit = 1 mm)	0.989 (0.975-1.004)	0.159	–	–	1.000 (0.983-1.016)	0.984	–	–
Long axis mm (continuous, unit = 1 mm)	0.986 (0.941-1.033)	0.549	–	–	1.026 (0.973-1.082)	0.340	–	–
Short axis mm (continuous, unit = 1 mm)	0.930 (0.859-1.008)	0.077	–	–	0.962 (0.878-1.054)	0.404	–	–
L/S ratio (continuous)	1.724 (1.162-2.559)	0.007*	1.810 (1.187-2.760)	0.006*	2.195 (1.421-3.390)	<0.001*	2.357 (1.458-3.812)	<0.001*
Fat (≥50% vs <50%)	1.286 (0.741-2.233)	0.371	–	–	1.497 (0.802-2.794)	0.205	–	–

SLNneg, tumor negative sentinel lymph node. DFS, disease free survival. OS, overall survival. HR, hazard ratio. CI, Confidence interval. L/S, long/short axis ratio.

p-values calculated with cox regression analysis. * Statistically significant p-values. -, non applicable in multivariate analysis.

^1^indicates history of cancer other than epithelial skin cancer. Complete table in **Supplementary Files**.

**Table 4:** Number at risk and events table overall survival.

	years	0	1	2	3	4	5	6	7	8	9	10
**L/S ratio** **≤ 1.9**	At risk	265	264	262	259	254	252	247	241	237	234	234
	Events	1	0	2	3	5	2	5	6	4	3	0
**L/S ratio** **> 1.9**	At risk	91	88	87	80	77	74	68	68	68	68	68
	Events	1	2	1	7	3	3	6	0	0	0	0

**Table 5:** Number at risk and events table disease-free survival.

		0	1	2	3	4	5	6	7	8	9	10
**L/S ratio** **≤ 1.9**	At risk	265	259	254	247	243	241	233	226	220	216	216
	Events	1	5	5	7	4	2	8	7	6	4	0
**L/S ratio** **> 1.9**	At risk	91	88	83	76	73	71	66	66	64	64	64
	Events	1	2	5	7	3	2	5	0	2	0	0

There was no association between OS or DFS and surface area, circumference, length of long or short axis and presence of more than 50% fat in the nodes. No significant associations were found between SLNneg morphometric measurements and survival in DCIS cases, **Supplementary Table 7**.

## Discussion

In routine clinical practice, lymph node (LN) staging is essential for estimating prognosis and determining therapy options for patients with breast cancer (BC). Whilst patients with LN metastasis are known to have poorer survival, variation in overall survival (OS) and disease-free survival (DFS) has been noted in BC patients without evidence of LN metastasis (5). One of the potential underlying mechanisms could be a variation in host anti-tumor immune response. It is recognized that LN size and cellularity can change in an inflammatory state (19). We hypothesized that tumor draining LNs will respond similarly as part of the host anti-tumor immunoreaction. The morphometries of tumor negative SLNs (SLNnegs) from cases with ductal carcinoma *in situ* (DCIS) or invasive breast cancer (iBC) were determined and withheld surface area, circumference, long axis, short axis and long to short axis (L/S) ratio.

The SLN is the first tumor draining LN and is essential in generating anti-tumor immune responses. Therefore, the SLNneg could reveal the status of the host anti-tumor immune response without the possible impact of metastatic tumor cells. We hypothesized that a favorable host anti-tumor immune reaction is reflected in the SLNneg and can be quantified with histopathological morphometry in relation to improved survival outcome. In this study, we demonstrated a relationship between high long axis to short axis (L/S) ratio (>1.9) of SLNneg and worse OS and DFS in iBC cases. These results suggests SLNneg shape is related to immunological functioning of the SLN.

To the best of our knowledge, there is no previous report using histopathological morphometries of SLNneg in BC in correlation to patient prognosis. Previous studies have used the size of lymph node axis or calculated L/S ratio predominantly to assess the likelihood of LN metastasis. A study using *in vivo* ultrasound of axillary LNs suggested L/S ratio below 1.5 to be indicative for presence of metastasis (20). Evaluation of axillary LNs with MRI and subsequent histopathological measurements of LNs from the axilla suggested that L/S ratio of less than 1.6 in combination with a length of the long axis of 10 mm or more to be the most accurate criteria for predicting presence of LN metastases (21). In our study 52% of SLNneg had a L/S ratio below 1.6 and still 30% of cases had a L/S ratio below 1.6 combined with a long axis over 10mm. This data suggests that factors other than presence of metastasis influence SLN morphometry.

It is well recognized that LN size and cellularity can change in LNs draining inflamed regions (19). In this study, it was hypothesized that SLNneg size is a surrogate marker for an active host anti-tumor reaction and hence increased SLNneg size is associated with favorable outcome (13). Based on the results of our study, there was no linear relationship between survival and SLNneg surface area, long axis length or short axis length. More research is needed to confirm our findings and evaluate underlying biological mechanisms. Moreover, this study showed no relation between SLNneg morphometries and survival within DCIS cases. This could be due to the low number of cases and low number of events within this group.

A lower L/S ratio, reflecting a rounder SLNneg, was related to high TILs. This suggests round shaped SLNneg resembles better immunological functioning compared to elongated SLNneg. In our study, high TILs were not associated with improved survival, which could be due to the high proportion of cases with ER positive BC. In ER positive HER2 negative BC, TILs are not found to be prognostic, which is in contrast with other BC subtypes (7). Regarding the high proportion of ER positive BC cases within this study, the importance of these findings should be emphasized since the role of the immune response in ER positive BC is largely unknown. SLN morphometry might give more elaborate information about host immune status in this BC subtype compared with TILs in the primary tumor.

A higher body mass index (BMI) and SLN fat content over 50% were associated with a larger surface area, long axis, short axis and age >55 years. Higher BMI was associated with a larger surface area of the SLNneg. These findings support those from a previous imaging study which also suggested a relationship between size of axillary LNs and BMI (22). In obese mice it is shown that impaired immune functioning and decreased lymphatic fluid transport were associated with fat deposition within LNs (23). However in this study BMI was not associated with the presence of >50% intranodal fat. This could be due to the chosen cutoff of the nodal fat percentage, or could suggest that there may be more factors than BMI influencing fat deposition in LNs. In our study, high intranodal fat was associated with age over 55 years, possibly indicating age related atrophy, also referred to as lipomatous atrophy of LNs (24). Further work is necessary to demonstrate whether increased fat content within LNs might be used as a biomarker of impaired LN functioning in humans. The association between enlarged LNs and increased intranodal fat, as demonstrated in the current study, confirms previous studies in BC patients (25). Although SLNneg with >50% fat content had a significant larger surface, circumference, long axis and short axis compared to non-fatty SLNneg, the L/S ratio was similar to non-fatty SLNneg. This indicates that the overall shape and proportions of SLNneg were not altered due to increased presence of fat.

The current study has some limitations. Due to the retrospective design of this study, detailed macroscopic information about the SLNnegs was not available. We assumed that smaller LNs were embedded intact and that nodes thicker than 5mm were transected through the hilus in the largest plane. The cut-off of 50% for nodal fat percentage is arbitrary, and future studies should objectively evaluate fat percentage and distribution in LNs. To confirm our findings, future research should validate SLNneg morphometry in other BC populations. Despite these limitations, our study is the first to suggest an association between an elongated SLNneg shape, defined as SLNneg with a L/S ratio greater than 1.9, and worse DFS and OS in all subtypes of iBC. Strengths of this study include the large cohort size with a long term follow up. Furthermore, SLNneg morphometries were created with software without knowledge of pathological or clinical data.

The observations in our studies could have potential clinical implications. Immunoreactivity characteristics in tumor draining LNs might serve as an indicator of immunocompetence of the patient. These new findings grant the opportunity for a pathologist to give additional prognostic information on the SLN without metastasis.

## Conclusions

Though patients with LN negative BC have good predicted survival, differences in survival can still be found, even when corrected for known prognostic clinicopathological factors and treatment modalities. This difference could be explained by variability in immune competence of the tumor draining LNs. In the present study, novel morphological characteristics of SLNneg were found to predict survival in iBC, possibly either reflecting or causing differences in immune response to the primary tumor. Elongated SLNneg shape, marked as a higher SLNneg L/S ratio, was significantly associated with worse DFS and OS in iBC. We demonstrated that SLNneg with a round shape is associated with high TILs in the primary tumor. Our findings suggest that SLNneg shape is an additional diagnostic biomarker in the prediction of survival in iBC patients. This is particularly of benefit for patients with hormone receptor positive BC, for whom the prognostic meaning of TILs is more insecure. Enhanced immunostaging techniques could help identify a subset of patients who might benefit from further treatment or downscaling of treatment.

## Data availability statement

The raw data supporting the conclusions of this article will be made available by the authors, without undue reservation.

## Ethics statement

The studies involving humans were approved by METC 2021-2603, medical-ethical testing committee Maastricht University Medical center+. The studies were conducted in accordance with the local legislation and institutional requirements. The ethics committee/institutional review board waived the requirement of written informed consent for participation from the participants or the participants’ legal guardians/next of kin because Retrospective cohort study without direct consequences to the patient.

## Author contributions

LK: Conceptualization, Data curation, Formal Analysis, Investigation, Methodology, Visualization, Writing – original draft, Writing – review & editing. SD: Conceptualization, Data curation, Formal Analysis, Investigation, Methodology, Visualization, Writing – original draft, Writing – review & editing. SK: Data curation, Formal Analysis, Methodology, Software, Supervision, Validation, Writing – review & editing. AH: Project administration, Resources, Supervision, Writing – review & editing. MS: Conceptualization, Investigation, Methodology, Resources, Supervision, Writing – review & editing. HG: Conceptualization, Formal Analysis, Investigation, Methodology, Resources, Software, Supervision, Validation, Writing – review & editing.
