# Supplementary material for: The prognostic value of the histological shape of tumor negative sentinel nodes in breast cancer
Source: Front Immunol. 2023 Oct 30;14:1258641. doi: 10.3389/fimmu.2023.1258641 (PMC10642264; doi:10.3389/fimmu.2023.1258641)
Supplement: Supplementary file 1 [file Table_1.docx]

**Supplementary table 1. STROBE guidelines**

|  | Item No | Recommendation |
| --- | --- | --- |
| **Title and abstract** | 1 | (*a*) Indicate the study’s design with a commonly used term in the title or the abstract |
|  |  | (*b*) Provide in the abstract an informative and balanced summary of what was done and what was found |
| **Introduction** |  |  |
| Background/rationale | 2 | Explain the scientific background and rationale for the investigation being reported |
| Objectives | 3 | State specific objectives, including any prespecified hypotheses |
| **Methods** |  |  |
| Study design | 4 | Present key elements of study design early in the paper |
| Setting | 5 | Describe the setting, locations, and relevant dates, including periods of recruitment, exposure, follow-up, and data collection |
| Participants | 6 | (*a*) Give the eligibility criteria, and the sources and methods of selection of participants. Describe methods of follow-up |
|  |  | (*b*) For matched studies, give matching criteria and number of exposed and unexposed |
| Variables | 7 | Clearly define all outcomes, exposures, predictors, potential confounders, and effect modifiers. Give diagnostic criteria, if applicable |
| Data sources/ measurement | 8* | For each variable of interest, give sources of data and details of methods of assessment (measurement). Describe comparability of assessment methods if there is more than one group |
| Bias | 9 | Describe any efforts to address potential sources of bias |
| Study size | 10 | Explain how the study size was arrived at |
| Quantitative variables | 11 | Explain how quantitative variables were handled in the analyses. If applicable, describe which groupings were chosen and why |
| Statistical methods | 12 | (*a*) Describe all statistical methods, including those used to control for confounding |
|  |  | (*b*) Describe any methods used to examine subgroups and interactions |
|  |  | (*c*) Explain how missing data were addressed |
|  |  | (*d*) If applicable, explain how loss to follow-up was addressed |
|  |  | (*e*) Describe any sensitivity analyses |
| **Results** |  |  |
| Participants | 13* | (a) Report numbers of individuals at each stage of study—eg numbers potentially eligible, examined for eligibility, confirmed eligible, included in the study, completing follow-up, and analysed |
|  |  | (b) Give reasons for non-participation at each stage |
|  |  | (c) Consider use of a flow diagram |
| Descriptive data | 14* | (a) Give characteristics of study participants (eg demographic, clinical, social) and information on exposures and potential confounders |
|  |  | (b) Indicate number of participants with missing data for each variable of interest |
|  |  | (c) Summarise follow-up time (eg, average and total amount) |
| Outcome data | 15* | Report numbers of outcome events or summary measures over time |
| Main results | 16 | (a) Give unadjusted estimates and, if applicable, confounder-adjusted estimates and their precision (eg, 95% confidence interval). Make clear which confounders were adjusted for and why they were included |
|  |  | (b) Report category boundaries when continuous variables were categorized |
|  |  | (c) If relevant, consider translating estimates of relative risk into absolute risk for a meaningful time period |
| Other analyses | 17 | Report other analyses done—eg analyses of subgroups and interactions, and sensitivity analyses |
| Discussion | | |
| Key results | 18 | Summarise key results with reference to study objectives |
| Limitations | 19 | Discuss limitations of the study, taking into account sources of potential bias or imprecision. Discuss both direction and magnitude of any potential bias |
| Interpretation | 20 | Give a cautious overall interpretation of results considering objectives, limitations, multiplicity of analyses, results from similar studies, and other relevant evidence |
| Generalisability | 21 | Discuss the generalisability (external validity) of the study results |
| Other information | | |
| Funding | 22 | Give the source of funding and the role of the funders for the present study and, if applicable, for the original study on which the present article is based |

**Supplementary Table 2. SLNneg morphometries and clinicopathological characteristics.**

|  |  | n | Surface area | | Circumference | | Long axis | | Short axis | | L/S ratio | |
| --- | --- | --- | --- | --- | --- | --- | --- | --- | --- | --- | --- | --- |
|  |  |  | mean mm2± SD | p-value | mean mm  ± SD | p-value | mean mm  ± SD | p-value | mean mm  ± SD | p-value | Mean ± SD | p-value |
| Age | <55y | 134 | 82.6 ± 52.3 | 0.312 | 41.8 ± 16.2 | 0.184 | 13.3 ± 4.8 | 0.647 | 8.6 ± 3.1 | 0.096 | 1.6 ± 0.5 | 0.140 |
|  | ≥55y | 289 | 76.9 ± 55.1 |  | 39.6 ± 16.1 |  | 13.1 ± 4.9 |  | 8.0 ± 3.0 |  | 1.7 ± 0.5 |  |
| Subtype | DCIS | 67 | 74.4 ± 50.7 | 0.488 | 40.4 ± 15.6 | 0.954 | 13.0 ± 4.8 | 0.783 | 7.9 ± 2.6 | 0.447 | 1.7 ± 0.5 | 0.654 |
|  | Invasive | 356 | 79.5 ± 54.9 |  | 40.3 ± 16.3 |  | 13.2 ± 4.9 |  | 8.3 ± 3.1 |  | 1.7 ± 0.5 |  |
| Side | Right | 212 | 79.3 ± 56.0 | 0.821 | 40.4 ± 16.8 | 0.845 | 13.2 ± 5.2 | 0.736 | 8.1 ± 2.9 | 0.700 | 1.7 ± 0.5 | 0.995 |
|  | Left | 211 | 78.1 ± 52.6 |  | 40.1 ± 15.6 |  | 13.1 ± 4.5 |  | 8.3 ± 3.1 |  | 1.7 ± 0.6 |  |
| BMI | ≤25 | 138 | 69.3 ± 42.5 | 0.003* | 38.9 ± 16.3 | 0.070 | 12.5 ± 4.7 | 0.014* | 7.8 ± 2.7 | 0.024* | 1.7 ± 0.6 | 0.824 |
|  | 26-30 | 113 | 87.4 ± 58.4 |  | 41.2 ± 15.1 |  | 13.6 ± 4.9 |  | 8.6 ± 3.2 |  | 1.7 ± 0.6 |  |
|  | >30 | 60 | 94.5 ± 65.6 |  | 44.5 ± 17.0 |  | 14.7 ± 5.1 |  | 8.9 ± 3.3 |  | 1.7 ± 0.4 |  |
| SLN Fat content | <50% | 350 | 69.1 ± 43.1 | <0.001* | 38.7 ± 15.8 | <0.001* | 12.5 ± 4.6 | <0.001* | 7.8 ± 2.8 | <0.001* | 1.7 ± 0.5 | 0.526 |
|  | ≥50% | 73 | 124.4 ± 75.2 |  | 47.6 ± 16.2 |  | 16.1 ± 5.4 |  | 10.1 ± 3.4 |  | 1.6 ± 0.5 |  |

SLNneg = tumor negative sentinel lymph node. BMI = body mass index. L/S ratio = long/short axis ratio. y = years. p-values calculated with One-Way ANOVA. *Statistically significant p-values.

Patients with a BMI ≤25 showed a significant lower SLN surface area than the other groups. Both long axis and short axis were significantly longer in patients with BMI> 30 compared to those with BMI ≤25. (GLM, Bonferroni).

**Supplementary** **table 3. Relationship between SLNneg morphometries of patients with invasive breast cancer and primary breast cancer characteristics.**

|  |  | Surface | | Circumference | | Long axis | | Short axis | | L/S ratio | |
| --- | --- | --- | --- | --- | --- | --- | --- | --- | --- | --- | --- |
|  | n | mean mm2  ± SD | p-value | mean mm ± SD | p-value | mean mm  ± SD | p-value | mean mm  ± SD | p-value | Mean  ± SD | p-value |
| TILs <10% TILs ≥10 | 257  95 | 76.9 ± 51.7  86.6 ± 62.5 | 0.139 | 40.1 ± 16.2  40.7 ± 16.8 | 0.760 | 13.2 ± 4.9  13.0 ± 4.9 | 0.732 | 8.0 ± 2.9  8.8 ± 3.5 | 0.042* | 1.7 ± 0.5  1.5 ± 0.4 | 0.005* |
| T1  T2^1^ | 283  67 | 78.3 ± 52.8  79.9 ± 57.8 | 0.825 | 40.5 ± 16.2  38.4 ± 16.5 | 0.329 | 13.2 ± 4.9  12.7 ± 5.1 | 0.425 | 8.2 ± 3.0  8.2 ± 3.2 | 0.949 | 1.7 ± 0.5  1.6 ± 0.5 | 0.270 |
| NST  ILC  Other | 266  28  58 | 79.1 ± 54.4  81.0 ± 52.3  81.9 ± 60.1 | 0.931 | 40.1 ± 16.7  36.8 ± 15.3  40.0 ± 15.0 | 0.456 | 13.3 ± 5.0  12.8 ± 5.1  13.0 ± 4.5 | 0.834 | 8.3 ± 3.0  7.9 ± 2.9  8.5 ± 3.4 | 0.663 | 1.7 ± 0.5  1.6 ± 0.5  1.6 ± 0.5 | 0.607 |
| Grade 1  Grade 2  Grade 3 | 99  149  102 | 79.9 ± 50.9  75.8 ± 55.4  85.4 ± 58.7 | 0.399 | 40.6 ± 14.3  39.0 ± 16.7  42.2 ± 17.8 | 0.325 | 13.1 ± 4.4  13.0 ± 5.2  13.5 ± 5.1 | 0.755 | 8.5 ± 3.1  7.8 ± 3.0  8.7 ± 3.2 | 0.059 | 1.6 ± 0.6  1.7 ± 0.5  1.6 ± 0.5 | 0.233 |
| ER neg  ER pos | 59  286 | 82.4 ± 63.5  79.0 ± 53.5 | 0.666 | 39.1 ± 17.5  40.6 ± 16.2 | 0.537 | 12.7 ± 5.4  13.3 ± 4.8 | 0.425 | 8.4 ± 3.4  8.2 ± 3.1 | 0.736 | 1.6 ± 0.5  1.7 ± 0.5 | 0.094 |
| PR neg  PR pos | 111  233 | 75.4 ± 53.6  81.8 ± 56.0 | 0.314 | 38.1 ± 15.7  41.5 ± 16.7 | 0.080 | 12.4 ± 4.8  13.6 ± 5.0 | 0.044* | 8.0 ± 3.0  8.4 ± 3.2 | 0.231 | 1.6 ± 0.5  1.7 ± 0.6 | 0.126 |
| HER2 neg  HER2 pos | 285  38 | 81.2 ± 56.2  74.3 ± 58.4 | 0.483 | 40.5 ± 16.6  40.6 ± 18.0 | 0.988 | 13.2 ± 4.9  12.9 ± 5.1 | 0.726 | 8.4 ± 3.1  8.0 ± 3.4 | 0.440 | 1.6 ± 0.5  1.7 ± 0.6 | 0.356 |
| Non TNBC  TNBC | 299  43 | 79.7 ± 55.0  80.6 ± 57.8 | 0.914 | 40.7 ± 16.4  38.4 ± 16.8 | 0.387 | 13.3 ± 4.9  12.7 ± 5.4 | 0.464 | 8.3 ± 3.1  8.2 ± 3.0 | 0.900 | 1.7 ± 0.5  1.6 ± 0.5 | 0.215 |
| Non LVSI  LVSI | 204  35 | 85.8 ± 56.8  69.6 ± 48.3 | 0.113 | 41.5 ± 15.1  39.8 ± 21.2 | 0.574 | 13.8 ± 5.0  12.9 ± 5.5 | 0.371 | 8.5 ± 3.0  7.9 ± 3.2 | 0.269 | 1.7 ± 0.5  1.7 ± 0.6 | 0.682 |

SLNneg = tumor negative sentinel lymph node. SD = standard deviation. T = tumor stage. NST = invasive breast cancer no special type. ILC = invasive lobular carcinoma. TNBC = triple negative breast cancer. LVSI = lymphovascular space invasion

p-values calculated with One-Way ANOVA. * Statistically significant p-values.

^1^ T3 patients excluded because of low number (n=2).

**Supplementary** **table 4. Relationship between SLNneg morphometries of patients with DCIS and its characteristics.**

|  |  | Surface | | Circumference | | Long axis | | Short axis | | L/S ratio | |
| --- | --- | --- | --- | --- | --- | --- | --- | --- | --- | --- | --- |
|  | n | mean mm2  ± SD | p-value | mean mm  ± SD | p-value | mean mm  ± SD | p-value | mean mm  ± SD | p-value | Mean  ± SD | p-value |
| Size ≤40mm  Size >40mm | 41  26 | 71,4 ± 51.0  79.2 ± 50.8 | 0.542 | 38.8 ± 13.8  42.9 ± 18.0 | 0.290 | 12.7 ± 4.6  13.5 ± 5.1 | 0.484 | 7.7 ± 2.5  8.3 ± 2.6 | 0.418 | 1.7 ± 0.5  1.7 ± 0.6 | 0.997 |
| Grade 1  Grade 2  Grade 3 | 6  20  41 | 55.2 ± 29.6  85.3 ± 61.6  72.0 ± 46.9 | 0.396 | 31.5 ± 11.1  41.0 ± 14.8  41.4 ± 16.3 | 0.347 | 10.4 ± 3.9  13.8 ± 5.1  13.0 ± 4.7 | 0.324 | 6.7 ± 1.3  8.2 ± 2.8  8.0 ± 2.6 | 0.467 | 1.5 ± 0.4  1.7 ± 0.4  1.7 ± 0.6 | 0.684 |

SLNneg = tumor negative sentinel lymph node. DCIS = ductal carcinoma in situ. SD = standard deviation.

p-values calculated with One-Way ANOVA.

**Supplementary table 5. Univariate and multivariate survival analyses of SLNneg morphometries in patients with invasive breast cancer.**

|  | Disease free survival | | | | Overall survival | | | |
| --- | --- | --- | --- | --- | --- | --- | --- | --- |
|  | Univariate | | Multivariate | | Univariate | | Multivariate | |
|  | HR (95% CI) | *p*-value | HR (95% CI) | *p*-value | HR (95% CI) | *p*-value | HR (95% CI) | *p*-value |
| Age  (continuous, unit = 1 year) | 1.060  (1.037-1.083) | <0.001* | 1.061  (1.037-1.086) | <0.001* | 1.076  (1.048-1.105) | < 0.001* | 1.072  (1.043-1.102) | <0.001* |
| TILs (continuous, unit = 1%) | 0.996 (0.982-1.011) | 0.625 | - | - | 0.998 (0.982-1.014) | 0.783 | - | - |
| TILs cut-off (<10% vs (≥10%) | 1.105 (0.667-1.829) | 0.699 | - | - | 0.756 (0.398-1.437) | 0.394 | - | - |
| Chemotherapy  (yes vs no) | 0.709  (0.397-1.267) | 0.246 | - | - | 0.916  (0.482-1.740) | 0.789 | - | - |
| Hormonal therapy  (yes vs no) | 0.671  (0.409-1.101) | 0.114 | - | - | 0.920  (0.526-1.608) | 0.769 | - | - |
| HER2-targeted therapy  (yes vs no) | 0.241  (0.033-1.733) | 0.157 | - | - | 0.352  (0.049-2.543) | 0.300 | - | - |
| Tumor size  (≥10mm vs < 10mm) | 1.471  (0.875-2.473) | 0.145 | - | - | 3.291  (1.487-7.281) | 0.003* | 3.134  (1.227-8.006) | 0.017* |
| Grade  (G3 vs G1-2) | 1.337  (0.831-2.151) | 0.232 | - | - | 1.342  (0.768-2.347) | 0.301 | - | - |
| Margin status  (negative vs positive) | 2.010  (0.494-8.190) | 0.330 | - | - | 2.764  (0.382-19.983) | 0.314 | - | - |
| Lymphovascular invasion  (yes vs no) | 1.473  (0.712-3.046) | 0.296 | - | - | 1.286  (0.533-3.106) | 0.576 | - | - |
| PR expression  (positive vs negative) | 0.523  (0.332-0.823) | 0.005* | 0.571  (0.317-1.026) | 0.061 | 0.524  (0.307-0.895) | 0.018* | 0.629  (0.301-1.315) | 0.218 |
| ER+/HER2-  HER2+  TNBC | Ref  1.118 (0.529-2.364)  1.969 (1.102-3.516) | Ref  0.769  0.022* | Ref  1.142 (0.513-2.541)  1.744 (0.839-3.624) | Ref  0.746  0.136 | Ref  1.195 (0.500-2.859)  2.249 (1.159-4.368) | Ref  0.688  0.017* | Ref  1.254 (0.472-3.334)  2.054 (0.851-4.958) | Ref  0.650  0.110 |
| Treatment adequacy (yes vs no) | 0.554 (0.335-0.916) | 0.021* | 0.727 (0.413-1.278) | 0.268 | 0.516 (0.288-0.925) | 0.026* | 0.819 (0.408-1.645) | 0.575 |
| Malignancy in history^1^  (yes vs no) | 2.328  (1.340-4.043) | 0.003* | 1.525  (0.831-2.796) | 0.173 | 2.638  (1.413-4.925) | 0.002* | 1.724  (0.847-3.512) | 0.133 |
| Surface mm^2^  (continuous, unit = 1 mm^2^) | 0.998  (0.993-1.002) | 0.298 | - | - | 1.001  (0.996-1.006) | 0.668 | - | - |
| Circumference mm  (continuous, unit = 1 mm) | 0.989  (0.975-1.004) | 0.159 | - | - | 1.000  (0.983-1.016) | 0.984 | - | - |
| Long axis mm  (continuous, unit = 1 mm) | 0.986  (0.941-1.033) | 0.549 | - | - | 1.026  (0.973-1.082) | 0.340 | - | - |
| Short axis mm  (continuous, unit = 1 mm) | 0.930  (0.859-1.008) | 0.077 | - | - | 0.962  (0.878-1.054) | 0.404 | - | - |
| L/S ratio  (continuous) | 1.724  (1.162-2.559) | 0.007* | 1.810  (1.187-2.760) | 0.006* | 2.195  (1.421-3.390) | <0.001* | 2.357  (1.458-3.812) | <0.001* |
| Fat  (≥50% vs less than 50%) | 1.286  (0.741-2.233) | 0.371 | - | - | 1.497  (0.802-2.794) | 0.205 | - | - |

SLNneg = tumor negative sentinel lymph node. DFS = disease free survival. OS = overall survival. HR = hazard ratio. CI = Confidence interval. L/S = long/short axis ratio.

p-values calculated with cox regression analysis. * Statistically significant p-values.

^1^ indicates history of cancer other than epithelial skin cancer.

**Supplementary table 6. Clinicopathological characteristics of invasive breast cancer cases stratified by SLNneg L/S ratio 1.9**

|  |  | L/S ratio ≤1.9 | | L/S ratio>1.9 | |  |
| --- | --- | --- | --- | --- | --- | --- |
|  |  | n | % | n | % | p-value |
| TILs | <10% | 178 | 69.3 | 79 | 30.7 | < 0.001* |
|  | ≥10% | 83 | 87.4 | 12 | 12.6 |  |
| T stage^1^ | T1 | 210 | 79.5 | 73 | 83.0 | 0.605 |
|  | T2 | 53 | 20.1 | 14 | 15.9 |  |
|  | T3 | 1 | 0.4 | 1 | 1.1 |  |
| Subtype^1^ | NST | 199 | 76.0 | 67 | 74.4 | 0.725 |
|  | ILC | 21 | 8.0 | 7 | 7.8 |  |
|  | Other | 42 | 16.0 | 16 | 17.8 |  |
| Grade^1^ | I | 78 | 30.0 | 21 | 23.3 | 0.604 |
|  | II | 105 | 40.4 | 44 | 48.9 |  |
|  | III | 77 | 29.6 | 25 | 27.8 |  |
| ER^1^ | Pos | 208 | 81.3 | 78 | 87.6 | 0.169 |
|  | Neg | 48 | 18.8 | 11 | 12.4 |  |
| PR^1^ | Pos | 167 | 65.2 | 66 | 75.0 | 0.091 |
|  | Neg | 89 | 34.8 | 22 | 25.0 |  |
| HER2^1^ | Pos | 27 | 11.1 | 11 | 13.9 | 0.495 |
|  | Neg | 217 | 88.9 | 68 | 86.1 |  |
| TNBC^1^ | Yes | 36 | 14.2 | 7 | 8.0 | 0.130 |
|  | No | 218 | 85.8 | 81 | 92.0 |  |
| Surgery | Lumpectomy | 151 | 57.0 | 54 | 59.3 | 0.695 |
|  | Mastectomy | 114 | 43.0 | 37 | 40.7 |  |
| Margin status | Negative | 253 | 95.5 | 86 | 94.5 | 0.710 |
|  | Positive | 12 | 4.5 | 5 | 5.5 |  |
| Radiotherapy^1^ | Yes | 154 | 58.6 | 52 | 57.1 | 0.815 |
|  | No | 109 | 41.4 | 39 | 42.9 |  |
| Systemic therapy^1^ | Yes | 117 | 44.5 | 48 | 53.3 | 0.147 |
|  | No | 146 | 55.5 | 42 | 46.7 |  |
| Therapy adequacy | Yes  No | 217  47 | 82.2  17.8 | 71  20 | 78.0  22.0 | 0.382 |
| Side | Right | 126 | 47.5 | 50 | 54.9 | 0.224 |
|  | Left | 139 | 52.5 | 41 | 45.1 |  |
| Malignancy in history^2^ | Yes | 28 | 10.6 | 14 | 15.4 | 0.220 |
|  | No | 237 | 89.4 | 77 | 84.6 |  |
| BMI | ≤25 | 93 | 49.7 | 31 | 43.7 | 0.135 |
|  | 26 - 30 | 60 | 32.1 | 23 | 32.4 |  |
|  | >30 | 34 | 18.2 | 17 | 23.9 |  |

SLNneg = tumor negative sentinel lymph node. L/S ratio = long/short axis ratio. NST = invasive breast cancer no special type. ILC = invasive lobular carcinoma. TNBC=triple negative breast cancer. BMI = body mass index.

p-values calculated with One-Way ANOVA. * Statistically significant p-values.

^1^ indicates that there were missing data but at least 90% data were available, except for BMI where only 72,5% of data were available.

^2^ indicates history of cancer other than epithelial skin cancer.

**Supplementary** **table 7. Survival analysis of SLNneg morphometries in DCIS cases.**

|  | Disease free survival | | Overall Survival | |
| --- | --- | --- | --- | --- |
|  | Univariate | | Univariate | |
|  | HR (95% CI) | *p*-value | HR (95% CI) | *p*-value |
| Age  (continuous, unit = 1 year) | 1.226 (1.037-1.450) | 0.017* | 1.228 (1.035-1.456) | 0.018* |
| DCIS size (≥40mm vs less than 40mm) | 5.136 (0.534-49.448) | 0.157 | 5.028 (0.523-48.361) | 0.162 |
| DCIS grade  (G3 vs G1-2) | 46.711 (0.012-181562.874) | 0.362 | 45.836 (0.011-182967.962) | 0.366 |
| Surface mm^2^  (continuous, unit = 1 mm^2^) | 0.987 (0.961-1.013) | 0.326 | 0.991 (0.996-1.017) | 0.489 |
| Circumference mm  (continuous, unit = 1 mm) | 0.993 (0.941-1.047) | 0.790 | 1.005 (0.953-1.060) | 0.848 |
| Long axis mm  (continuous, unit = 1 mm) | 0.934 (0.767-1.138) | 0.500 | 0.971 (0.796-1.185) | 0.774 |
| Short axis mm  (continuous, unit = 1 mm) | 0.848 (0.575-1.250) | 0.404 | 0.893 (0.598-1.333) | 0.579 |
| L/S ratio  (continuous) | 2.024 (0.501-8.179) | 0.323 | 2.822 (0.625-12.748) | 0.177 |
| Fat  (≥50% vs less than 50%) | 0.040 (0.000-1068.263) | 0.535 | 0.040 (0.000-2610.239) | 0.569 |

SLNneg = tumor negative sentinel lymph node. DCIS = ductal carcinoma in situ. DFS = disease free survival. OS = overall survival. HR = hazard ratio. CI = Confidence interval. L/S = long/short axis ratio. p-values calculated with cox regression analysis. * Statistically significant p-values.
